# The Examination of Medical Students

**Published:** 1896-06-20

**Authors:** 


### The Hospital

An Institutional Journal of

The Medical Sciences and Hospital Administration, with

AN EXTRA NURSING SUPPLEMENT.

Vol. XX.—No. 508] [June 20, 1896.

### The Examination of Medical Students.

There can be no doubt of the importance of the subject brought before the General Medical Council by Mr. Pridgin Teale in his notice of motion in regard to the number and extent of the examinations to which medical students are subjected, and by which, from his point of view, the study of medicine is retarded and interfered with.

By degrees the list of examinations has been so increased that practically students are never without the fear of one before their eyes, and thus, instead of being able to avail themselves of the enormous field of clinical observation offered by the hospitals, a field such as will never again be at the disposal of most of them, modern students neglect these great opportunities "because they dare not tear themselves away from the drudgery of book work, dreading failure in the examination." It is this displacement of clinical work by book knowledge which is the most serious result of the modern system so far as the progress of medicine is concerned, for it is notorious that the average newly-qualified man is lamentably lacking in practical acquaintance with much that he ought to know.

From the medical students' and parents' point of view also the system is equally unsatisfactory, for the large number of rejections at the final examination show that this method of harrying students all their time by repeated examinations renders it very difficult for even the best of them to make sure of getting through.

For whose benefit then is it maintained? The answer to this is: For the control of the schools and the governance of the unsatisfactory students. No doubt it was vexatious that a parent, after all the expense of paying fees, and keeping his son for four long years ostensibly as a medical student, should find out only at the end that all this time had been wasted in idleness or in dissipation, and it seemed obvious that if the examinations had but taken place at shorter intervals such a result could not have happened. As they said, the thing would have been put a stop to at once. So for the regulation of the unsatisfactory student, and to prevent him from wasting his father's substance in riotous living, the present system of constant examinations has been introduced, to the plague and annoyance of all the better men. How inoperative it has been, however, for all good purposes may be seen from the fact that the number of men rejected at the final increases year by year. Are we to believe that medical students are less industrious than they used to be, or that they give themselves up less fully to the work that is set before them? On the contrary, never did we have better men, never did we have men who gave themselves up more thoroughly to the work that is set them; but according to Mr. Teale this is the wrong sort of work, for instead of learning their profession they are learning to pass examinations so contrived as to prove that they have not been wasting their time but have gone stage by stage through the prescribed curriculum.

This is undoubtedly a wasteful process; wasteful of the student's time, wasteful of his energies, and especially wasteful of his opportunities—those great opportunities of clinical work, under the supervision and criticism of able teachers, which come to a man only while he is a student in the wards of a great hospital.

What Mr. Teale would have done, then, would be to relegate to the schools the governance of their own students. A curriculum having been laid down, it should be left to the authorities of the schools to see that it is properly followed, that each individual student goes through each course and attains a due proficiency in each method. The schools should, in fact, do in reality what they now profess to do when they sign for their students. The examining bodies then, being relieved of all responsibility as to the methods of teaching—having received from the college authorities satisfactory certificates that the student had been properly taught—would confine themselves to ascertaining that as a result he had a sufficient knowledge of those subjects which are required in his daily work as a practitioner of medicine, surgery, and midwifery. It is the principle of a teaching university over again; in which the professors' teaching and the students' work, and even the class result of that work, are certified by the college, and the central examining authority confines itself to ascertaining the final product.

Such a plan would, of course, involve placing a greater amount of confidence in the college authorities than probably many of them deserve, and would certainly involve a thorough system of visitation and inspection; but it must be remembered that a great deal of the present examination system has for its object the control of the schools as well as the discipline of the students, and it seems reasonable to believe that the best way of controlling the schools would be to examine them rather than their students. What the result of Mr. Pridgin Teale's motion will be no one can tell. The council adopted the not very logical course of referring this criticism of their examinations to their Examination Committee, and, like seed laid in somewhat arid soil, it will, no doubt for a time, be heard no more of. That in some form or other, however, it will ultimately bring forth fruit we cannot doubt. Examinations are part of modern life; they are the only substitute as yet discovered for favouritism and jobbery. But they should be devoted to proper ends and carried out in a proper manner. The end should be to ascertain whether a man who is guaranteed to have gone with credit through the arranged curriculum is fit to practise, and not, as at present, to ascertain whether or no he has gone with credit through the curriculum; and the manner should be slow, considerate, and thorough—not a scramble to the tune of a ten minutes' bell.

